# Cultural understanding, experiences, barriers, and facilitators of healthcare providers when providing preconception counseling to adolescent Latinas with diabetes

**DOI:** 10.7243/2054-9865-5-2

**Published:** 2018

**Authors:** Frances M. Peterson-Burch, Ellen Olshansky, Hiba A. Abujaradeh, Jessica J. Choi, Robynn Zender, Keirsten Montgomery, Amy Case, Dara H. Sorkin, Diego Chaves-Gnecco, Ingrid Libman, Candice Taylor Lucas, Frank Zaldivar, Denise Charron-Prochownik

**Affiliations:** 1 University of Pittsburgh School of Nursing, 440 Victoria Building, 3500 Victoria Street Pittsburgh, USA.; 2 Professor Emerita, University of California, Irvine Sue & Bill Gross School of Nursing 802 W Peltason Drive Irvine, CA 92697, USA.; 3 University of California, Irvine, Sue & Bill Gross School of Nursing, 802 W Peltason Drive Irvine, CA 92697, USA.; 4 CHOC Children’s Endocrine Division, 1201 W. La Veta Ave Orange, Ca, USA.; 5 Consortium for Independent Research, 512 E. 27th Street Vancouver, WA, USA.; 6 Department of Medicine 100 Theory, University of California, Irvine, Suite 110 Irvine, CA, USA.; 7 MD, MPH, FAAP UPMC Children’s Hospital of Pittsburgh, Oakland Medical Building, 3420 Fifth Ave. Pittsburgh, PA, USA.; 8 University of California, Irvine School of Medicine, 333 The City Blvd. West, Suite 800 Orange, CA, USA.; 9 Department of Pediatrics University of California, Irvine, Pediatric Exercise and Genomics Research Center (PERC), UC Irvine School of Medicine, 101 Academy, Suite 150 Irvine, CA, USA.

**Keywords:** Preconception counseling, adolescents, diabetes, Latinas, reproductive health

## Abstract

**Background:**

Latinas are at a higher risk than Caucasians for both type 1 and type 2 diabetes (DM), as well as DM-associated reproductive health (RH) complications. Healthcare providers (HCPs) should deliver culturally-sensitive care to enhance the care relationship between Latinos and HCPs and to improve patient outcomes. This study explored an expert panel’s cultural understanding, experiences, barriers, and facilitators regarding RH and preconception counseling (PC) for adolescent Latinas with DM and their families.

**Methods:**

This study used open-ended questions with a focus group of 8 HCPs from the mid-Atlantic, Southwest, and Northwest regions of the United States in a teleconference format. Two researchers transcribed and reviewed the transcript for accuracy. Using content analysis, four members of the team identified themes. All researchers discussed themes and a 100% consensus was reached. For confirmation, a coding protocol was created based on the emerging themes.

**Results:**

Five themes related to cultural understanding and experiences were identified: 1) issues of identity; 2) acculturation; 3) stigma; 4) ambivalence toward birth control, RH education, and PC; and 5) cultural sensitivity vs. best practice. Four barriers were identified: 1) language; 2) religion; 3) access to healthcare, and 4) discomfort with discussion. Ten facilitators were identified: 1) the importance of support and support networks; 2) promoting trust among HCPs, daughters, and families; 3) assessing emotional development; 4) empowerment; 5) emphasizing safety; 6) communicating in patients’ preferred language; 7) discussing RH-related topics and PC using cultural sensitivity; 8) importance of being ready/temporality/planning for the future; 9) the importance of family-centered care; and 10) variation in educational tailoring and dissemination/ care delivery.

**Conclusions:**

Findings support the need for culturally sensitive and developmentally appropriate PC programs to empower adolescent Latinas with DM.

## Introduction

Diabetes mellitus (DM) is a contributing factor to reproductive health (RH) complications, prenatal and neonatal complications, and maternal/child mortality [1]. Uncontrolled DM can increase the risk of maternal/child complications, including spontaneous abortion, fetal anomalies, preeclampsia, fetal death, and microsomia [1]. To mitigate these risks, the American Diabetes Association recommends that all women with DM who are of childbearing age receive preconception counseling (PC) beginning at puberty to decrease the risk of adverse outcomes [1].

Both type 1 and type 2 DM are increasing in Latino youth [2,3]. In Latina adolescents aged 15–19, the incidence of type 2 DM exceeds that of type 1 DM [3], and increased rates of poor glycemic control, dyslipidemia, and overweight and obesity among young Latinos under age 19 may put them at a higher risk for future DM-related complications [3,4], including retinopathy, cardiovascular disease, and renal damage [1]. The increased frequency of all types of DM among Latinas in their childbearing years also impacts future generations’ risk, as intrauterine exposure to maternal DM and maternal obesity increases the risk of type 2 DM in the children [5,6]. Women who have DM during pregnancy give birth to children who are also at an elevated risk of early-onset DM, which leads to a vicious cycle of disease risk [7]. A further consideration is the high rate of teen births amongst Latinas, who have twice the rate of live births than those of non-Latino whites [8], are more likely to be younger (13–15 years of age) [9], and less likely to have taken periconceptional folic acid [9].

A key element in preventing RH complications, unintended pregnancy, and poor maternal/child outcomes in the context of DM is for HCPs to provide PC during routine clinic visits. PC is personalized advice, information and care from a specialized team of health professionals to help women with DM achieve optimal glycemic control prior to pregnancy and delay future pregnancies until it is medically safe, decreasing the risk of complications [1]. While PC has been proven to be cost-effective and efficacious in improving knowledge, family planning vigilance, and optimal RH behaviours [10–13], it is often not initiated before sexual debut, which is a critical time to deliver information to prevent risky sexual behavior and DM-related RH complications. HCPs must be aware of culturally-sensitive ways to provide PC and RH information to optimize the RH outcomes of Latina adolescents with DM.

The objective of this study was to explore issues raised by an 8-member HCP panel regarding cultural understanding/knowledge, experiences, barriers, and facilitators when providing PC and RH information to adolescent Latinas with DM and their families.

## Materials and Methods

This qualitative study used a tele-conference focus group of eight HCPs from the mid-Atlantic, Southwest, and Northwest regions of the United States. The tele-conference was conducted in 2015. The HCPs consisted of two pediatricians, two pediatric endocrinologists, a public health expert, a pediatric nurse practitioner, a pediatric psychologist, and an exercise-focused immunologist. The HCPs were recruited via convenience sampling and were selected due to the frequency in which they provided health care for Latinos. Their workplaces include children’s hospitals, outpatient DM clinics, laboratories, and a Spanish-speaking pediatric outpatient clinic. The HCPs accepted patients with private insurance, Medicaid, and/or lack of insurance. Seven out of the eight HCPs were affiliated with an academic center. Two panelists identify as Latino, and the remaining HCPs frequently care for the Latino population. Four indicated that they had the ability to speak Spanish in the clinical setting. Two HCPs were male and six were female. All providers were experts in DM, adolescents, PC, and/or RH.

The interview guide was based on the Expanded Health Belief Model, a theoretical model that incorporates a process orientation to explore patients’ beliefs on preventative healthcare management and adds several constructs to explain difficulties practitioners encounter in fostering realization of health risks and/or gaining long-term compliance with preventive health care recommendations [14]. It is particularly useful for focus group research [14]. Open-ended questions addressed cultural understanding/knowledge, experiences, facilitators and barriers to delivering information on DM-specific RH information and PC to adolescent Latinas and their families (Table 1). The conference call was recorded and transcribed verbatim. Two members of the research team reviewed the transcript for accuracy. Using content analysis, four members of the research team analyzed the data and identified themes that reflected the understanding and experiences of the HCPs, focusing on the barriers and facilitators. All members discussed the themes and a 100% consensus was reached. For confirmation, a coding protocol was created based on the emerging themes. One researcher applied the codes to the transcript using ATLAS.ti software (Version 7).

## Results

### Cultural Understanding/Knowledge and Experiences

The HCP panel shared culturally-relevant knowledge and experiences that can impact DM-specific PC and RH care for adolescent Latinas with DM. Their perceptions are described below, organized according to the salient themes that were generated by the content analysis (See Table 2 for quotes).

Issues of Identity– “Latino youth want to blend in with their peers. The last thing that they want to do is stand out.”

Many of the HCPs indicated that their adolescent Latina patients, like other adolescents, place a high priority on conforming to peers. The HCPs state that the girls want to resemble other American adolescents and do not want their ethnicity or DM diagnosis to mark them as being different. Conversely, HCPs felt that mothers feel more comfortable with their Latino heritage and may value being different. The HCPs felt that the mothers do not place the same amount of importance on blending in with American culture as their daughters do, which can exacerbate the stress the daughters face about their own identities.

Acculturation--”Acculturation is more than generational differences. The longer you have been in this country, the higher the chance of acculturation.”

Closely related to identity is the issue of acculturation, which the HCPs perceived as having distinct generational differences. For example, the HCPs stated that adolescent Latina daughters prefer to speak English rather than Spanish. The HCPs stated that many first-generation parents almost always prefer to speak only Spanish and often choose specialty Latino clinics, adding further stress to the daughters’ desire of an American-acculturated identity.

The HCPs perceived that acculturation also plays a role during clinic visits. They stated that some of the second-generation daughters prefer to interact with HCPs alone and are comfortable with this practice. Conversely, HCPs stated that many first-generation Latino parents appear to prefer to stay in the examination room during their daughters’ appointments. The HCPs reported that the parents are not used to the HCPs having medical discussions with the adolescents alone and are skeptical about the need for private conversations. The HCPs reported feeling tension between themselves and the parents when the HCP asks the parent for an opportunity to speak to the adolescent alone, and the parents’ refusal to leave sometimes prevents the HCP from having conversations regarding PC and RH with the adolescent. When PC-related topics are discussed, providers stated that there is often a stark difference between the way that second generation daughters view contraception and PC versus their first-generation parents and grandparents, which can make the conversation strained. Lastly, the HCPs felt that parents often seem comfortable bringing up other problems in their families’ lives that were unrelated to the clinic visit, while the adolescent Latina patients felt uncomfortable doing so until a stronger relationship was established with the HCP.

Stigma--”Teens face a lot of self-image challenges where they are comparing themselves to others. When you’re dealing with children who have obesity, they may often be bullied or may be bullying others.”

The HCPs stated that they felt their adolescent Latina patients often struggle with the stigma of “otherness” that their DM diagnosis and the differing levels of acculturation between the adolescents and their parents sometimes present. HCPs believed that their adolescent Latina patients struggle with self-image, ethnic and cultural “otherness”, identity issues related to their DM diagnosis and body (especially if they are obese), and negative appraisals when comparing themselves with other adolescents. In addition, HCPs felt that stigma and its surrounding stress could result in the adolescents being bullied or in bullying others.

Ambivalence toward Birth Control Use, RH Education, and PC Knowledge--”I think one of the stark differences between Latino populations and other populations is the lack of wanting to use birth control. There are thoughts that are not non-accurate but that may not be as accurate with the current medical understanding of how contraception works.”

Our panel stated that some adolescent Latinas and their parents, particularly those who are first-generation, are very reluctant and resistant to discuss PC and RH with providers. The HCPs felt that some second-generation Latina adolescents’ reluctance was related to the parental presence in the room. In addition, both the adolescent Latinas and their parents were impacted by the barriers that are described in detail in the next section of this article. The HCPs stated that it is not unusual for families to refuse to have RH-related conversations by stating that the daughter is not sexually active and therefore the information is not necessary. In addition to the resistance to using birth control, HCPs perceived generational and acculturation differences in the way that birth control works, when it should be used, and when it is appropriate to begin to plan for childbearing.

Struggle Between Cultural Sensitivity and Best Practice--”Introducing contraception for the management of polycystic ovarian syndrome or a heavy period—aside from family planning—is often cut off, especially based on a religious perspective.”

While the HCPs wanted to respect the cultural and generational differences present among daughters, mothers, and themselves, they cited the struggle between being sensitive to the needs of their patients and the need to employ best practice standards. This often came up when discussing PC and RH, especially when addressing the need for strategies to avoid unplanned pregnancies or the utilization of hormonal birth control for non-contraceptive reasons, such as the management of dysmenorrhea. This was particularly true if the families had low levels of education and/or were unaware of the therapeutic indications of contraceptives that extended beyond pregnancy prevention.

### Barriers to Providing DM-Specific PC and RH Information

The HCPs noted several barriers to providing PC and other RH-related information to adolescent Latinas with DM and their families (Table 3). These barriers are described below:

Language barriers – “If they can relate to you, especially if you speak the same language, adherence is better.”

HCPs indicated that, unlike their daughters, many of their patients’ parents prefer to, or were only able to, communicate in Spanish. This could cause confusion or impact the quality of care and the patient-provider relationship, especially if the provider was not bilingual. Some HCPs stated that language barriers were related to the first-generation Latino family’s ability to relate to or trust the provider, which then could affect the adolescent’s adherence to the DM treatment plan.

Impact of Religion--”Religion plays a role. The parents and grandparents follow religion a little bit more. They’re a little bit more aware of what the church may say, while the younger generations are a little bit more open. I’ve had instances of conflict when you try to talk about sexual and reproductive health.”

The HCPs indicated that religion impacts the Latina adolescent’s decision to use contraception and what methods were frequently employed. They felt that these religious differences are more pronounced amongst many individuals who are first-generation, rather than those who are second-generation or beyond. HCPs stated that first-generation patients are less likely to use hormonal contraceptives, citing religious beliefs. Some HCPs felt constrained by the parents’ religious beliefs and felt that the impact of religion altered patient education, the quality of patient interactions, and the visit outcomes.

Access to Healthcare--”Many Latina youth experience barriers to healthcare access, insurance, etc.”

Our HCPs stated that healthcare coverage and access to care are not always available to their Latina patients and families, which could affect their health outcomes. Some patients, particularly first-generation immigrant parents, preferred to seek out specialty Spanish-speaking clinics to receive necessary services. However, HCPs perceived that seeking out Spanish-speaking specialty clinics was often in conflict with some adolescent Latinas’ issues of identity and acculturation. The HCPs felt that some adolescent Latinas who had a higher degree of acculturation felt that going to a Spanish speaking clinic made them stand out as an “other” for being both Latina and for having DM, which was in conflict with their desire to blend in and be a “normal” American adolescent. Receiving services at a Spanish-speaking clinic, especially with their parents who may have lower levels of acculturation, made some adolescent Latinas with DM feel different than their peers, and the HCPs felt that many of the adolescent Latinas would prefer to seek care from non-Spanish speaking clinics.

Discomfort--”If the kid unfolds into a giggly mess, you should probably just move on at that point because the child and family are uncomfortable. “

HCPs stated that some families seem comfortable receiving PC and RH-related information, but that they felt that other families did not seem ready or open to the discussion. HCPs felt that it was often the parents’ discomfort, rather than the daughters’, that prevents the dissemination of RH-related conversations. HCPs felt that the family’s discomfort then caused the adolescent Latina to be uncomfortable. When both the adolescent and her family became uneasy, the HCPs often became uncomfortable with discussing these important topics, which could alter the quality and delivery of care. HCPs stated that they tend to be more comfortable talking about RH and PC with older teens (from ages 17 and up) than they are with younger teens (ages 13 to 16), although they usually started talking about sex when their patients were ages 13 to 15.

### Facilitators to Providing DM-Specific RH Information and PC Care

Several facilitators were noted in our discussion. These are described in the following section (Table 4).

The Importance of Support and Support Networks—”Using promotoras helps to promote trust. That is something that we continue to do and it seems like people respond to that.”

Some of our HCPs cited the positive influence of using support networks to enhance DM treatment adherence, educational efforts, and patient outcomes. These networks could include extended families, college students/peer coaches, school-based programs, *promotoras* (lay community workers,) and Latino peer coaches. In addition to potentially providing knowledge and education, these networks could increase the patient’s feelings of being supported and decrease the feelings of being stigmatized by their “otherness.”

Promoting Trust between HCPs, Daughters, and Family—”I think that trust and listening to not just the diabetes, but everything that goes with their social issues (is important).”

Trust was a major theme that was cited by our participants. HCPs felt that mutual trust between the HCP and the family, including mothers, fathers, and other relatives, was an important component of providing quality care. Our participants stated that receiving care from a HCP from the same cultural or ethnic background and communicating in their preferred language are major components in facilitating trust. In a similar way, HCPs stated that *promotoras* and other Latino healthcare advocates were effective in facilitating trust in this population. Our panel stated that while first-generation parents were more trusting of providers, especially those from the same cultural background, teens were reluctant to openly discuss their lives and bring up issues to any providers, regardless of if they were Latino or of another ethnicity, until they felt like they could establish trust.

Assessing Emotional Development--”Every child is different. Sometimes (medical) residents will come back from an assessment and say, ‘You know, I don’t know that I feel that comfortable talking about sex with this child because they seem so young,’ and I think that, at times, there’s the aspect of ‘Okay, so they may seem young, but they’ll still have questions,’ and the added aspect of ‘Maybe they seem young and really are too young to absorb this information.’”

The HCPs stated that the daughter’s developmental stage is very important in providing care and is sometimes independent of chronological age. HCPs stated that providers sometimes feel uncomfortable discussing PC and RH with teens who are perceived to be too young to handle the information, despite its relevance to DM. However, the HCPs emphasized that every patient is unique, and the variability in their adolescent patients’ maturity and readiness required flexibility and continued assessment on behalf of the providers.

Empowerment--(On making decisions that support a healthy pregnancy) “I think it’s very important that they feel empowered and making them feel that they have the power to decide these things and learn more.”

Providers talked about the importance of empowering emotionally mature patients who are willing to talk about PC and RH, especially in the context of DM, in order to optimize outcomes. Providers endorsed reassuring patients about the feasibility of having a healthy reproductive future by emphasizing that adolescents have the power to choose their RH outcomes and fertility plans and that empowerment is essential in healthy decision-making. The HCPs stated that helping the adolescent Latina with DM feel that she can make healthy RH-related choices and become educated about her options is an important component of providing PC to this population.

Emphasizing Safety--”Allow them to be safe with their diabetes and to think about the future so that it may motivate them to be safe with their diabetes.”

The concept of safety is also a theme in the conversations that HCPs had with their adolescent Latina patients with DM, although this can manifest in different ways. Some providers talked about the importance of allowing patients to feel safe in their bodies and circumstances, despite their DM diagnosis. For example, HCPs used PC information as a way to empower the teens to make healthy RH and DM-related choices and motivate them to be safe in the future. Other providers emphasized the importance of being safe and achieving tight glycemic control before and during a pregnancy to facilitate better outcomes for offspring of patients with DM.

Communicating in the Patients’ Preferred Language— “Sometimes, splitting and speaking in English to the adolescent and Spanish to the parent has been really helpful in facilitating the interrelationship with us as a team with the parent and the child.”

As noted, there was often a divide between daughters and their family members in what language was preferred. Participants pointed out that it was important not to assume that they knew which language would be most desirable, and that it was beneficial to ask the families about their preferences. Some HCPs stated that they use both English and Spanish during appointments to optimize communication between all parties based on their preferences. They believed that doing so promotes trust and often improves treatment adherence.

Discussing RH-Related Topics and PC in a Culturally Sensitive Manner--”Mention marriage. Find some way to work the story around marriage and sex and reproducing as part of a package.”

An awareness of cultural sensitivity was highly important when providing PC and RH information. Participants stated that PC and RH-related conversations sometimes cause conflict between the HCP and families, as some families feel that these discussions imply that the teen is already sexually active. In response, some families disengage from the conversation by stating that the teen is not having sex, and therefore, RH information and PC are unnecessary. However, our HCPs reported that many Latino families felt more comfortable with these discussions if they were part of a larger narrative that included the adolescent’s future marriage, sexual relationship with her husband, and children, and that the conversation was framed in a way that indicated that these events would happen in that order.

Importance of Being Ready/Temporality/Planning for the Future--”I say that we are giving this to you in preparation (for the future). It makes it easier for families to accept the information versus it being something like, ‘No, this doesn’t apply to her, because she’s not having sex.’”

HCPs stated that PC and RH-related discussions with adolescent Latinas and their families are greatly facilitated when introducing the idea that everyone is ready for sexuality at different times, and that the timeline may be different for the daughter than it was for her other family members. The HCPs often told the family that this information is being given in preparation for the future and not because they believe that the patient is currently sexually active. The HCPs also stated that PC-related conversations are much more palatable to families when providers emphasize that controlling DM prevents RH complications and protects future fertility when the adolescent is ready to start her family.

Importance of Family-Centered Care— “The more you involve the extended family, the better.”

HCPs stated that their Latino patients place high importance on family, and that many healthcare decisions are made by family members (including mothers, fathers, grandmothers, and other extended family), rather than solely by the adolescent daughter. Our HCPs emphasized that the perspectives of the extended family members carry weight and can impact decision outcomes. The HCPs stated that, in some families, grandparents-not the adolescent patient or her parents- are the primarily decision makers in the adolescent’s DM, PC, and/ or RH-related care. HCPs stated that while the adolescent’s parents may be present and involved in the discussion, the final decision often comes from the grandparents.

Many HCPs stated that mothers want to be fully involved with the adolescent daughter’s care. The influential but handsoff role of fathers was also cited. Several of our HCPs felt that the adolescent’s father, while almost always absent during clinical visits, was a primary figure whose perspective had to be considered, and that both the daughter and her mother must talk to the father about the proposed treatment plan before any decision could be made. However, HCPs felt that fathers did not want to know intimate details of their daughters’ RH-related information, as this often makes them feel uncomfortable.

Variation in Educational Tailoring and Dissemination/ Care Delivery—”I would break down (PC and RH information) into different sections over a year, but those with different educational levels may have a little trouble with it.”

Our participants noted that there were differences in the way that PC and RH information was comprehended, depending on the family’s education levels, acculturation, and background. Our HCPs stated that some aspects of care, such as pictures of bodily anatomy, were very offensive to many first-generation immigrants in a clinical setting. One HCP shared that some first-generation families complained to clinical administration about anatomical pictures depicting nudity and successfully requested that the pictures be covered with clothing. Other important considerations included religion, gender, region of origin, diaspora, and whether they came from a rural or a city origin. Some of our HCPs stated that patients could be overwhelmed by too much information at one visit, especially if the patient had lower levels of education.

## Conclusions and Implications

This study explored an expert panel’s cultural understanding, experiences, barriers, and facilitators regarding PC and RH in adolescent Latinas with DM and their families. Our findings suggest that cultural, religious, developmental aspects, and family support network are important to consider when providing PC and RH information to this population. Many of these themes were interrelated. Special care must be taken to avoid making assumptions about acculturation status and preferences of the patients, as this can greatly affect the quality of care. This information will be used to culturally tailor an existing PC educational program for this population.

Based on the information shared by the HCPs in this study and supported by the literature, we suggest the following clinical implications, organized according to concepts generated from the qualitative data:

Our results indicate that providers should avoid treating the adolescent Latina with DM as “different” or “unusual”, as this could also cause stigma. Our HCPS indicated that adolescent Latinas want to blend in with other peers and not stand out as being different due to their DM or ethnicity. Research indicates that DM-related stigma is associated with poorer glycemic control in adolescents among all ethnicities [15], and that having DM makes many adolescents feel less “normal” than those without DM [16]. One study found that over 68% of female adolescents with type 1 DM stated that they experienced a personal experience of stigma, and the odds of poor glycemic control were more than twice as high in adolescents who stated that they felt stigmatized compared to those who did not [15]. Our study indicates that HCPs should talk with their adolescent Latina patients about issues of self-image, developing identities, healthy body image, bullying, mental health concerns, and if feelings of stigma are affecting health behaviors and/or DM management. In addition, our study indicates that HCPs must be cognizant that the adolescent may not feel the same cultural ties to her Latino heritage as other family members and that it is important to not make assumptions about her feelings and decisions relating to PC, RH, and sexuality based on her ethnicity.

Our study also supports the importance of overcoming language barriers in the clinical setting. Research has shown that parental limited English proficiency negatively impacts healthcare quality for children with special healthcare needs and should be considered a risk factor for poorer outcomes [17]. Other studies have found that fewer cross-cultural experiences during training is associated with pediatric-focused HCPs feeling less prepared to care for patients with low English proficiency [18]. Evidence shows that parental limited English proficiency has been associated with decreased overall quality of life for children with DM [19]. To meet this challenge, our HCPs indicated that providers should assess which language the family prefers to speak. This may entail speaking English to the child and Spanish to the parents, if preferred, in order to provide quality care while honoring the child’s and parents’ acculturation and identity. Previous research suggests that adolescents are often asked to interpret during healthcare tasks, such as reading prescriptions and talking to doctors [20], which presents ethical issues. A professional translator may need to be utilized as a best practice method [21].

Our HCPs suggested that acculturation differences must be respected when providing care. Some HCPs suggested speaking with the adolescent alone and then with the family together, particularly if they have differing levels of acculturation. Speaking to the adolescent Latina alone honors her need for autonomy and identity while honoring the family-centered care approach that is preferred by some of the first-generation parents.

Previous research indicates that acculturation may affect RH-related outcomes. Studies suggests that some first-generation Latina women with low levels of acculturation were raised to believe that talking about sex is sinful [22], and thus may be more reluctant to receive RH-related education from providers. Many first-generation Latinas cite poor quality sex education in their countries of origin [22], which may help to explain some first-generation Latinas’ poor knowledge about RH, sexuality, RH services [23,24], and shame/discomfort when asking for contraception [22]. One study among Latina adolescents and young women indicated that individuals with low to middle levels of acculturation used less effective methods of contraception compared to those with higher levels of acculturation [25]. As supported by our study, the literature indicates that patterns of contraception use among first-generation Latina women remain lower than those born in the United States, even after accounting for marital status, socioeconomic status, health insurance coverage, and religiosity [26]. Because culture plays a critical role in cognition and behaviors related to DM, the American Association of Diabetes Educators (AADE) recommend the HCPs and the organization be culturally sensitive [27]. Similarly, our study suggests that culturally tailoring PC and RH education to Latino parents and their adolescent daughters with DM could be associated with better RH outcomes.

In order to be culturally sensitive while providing best practice, HCPs also need to be aware of the charged nature of PC and RH-related conversations. Our study found that explaining the HCP’s thought process when designing DM and RH-related treatment plans was helpful for facilitating the Latino family’s comfort and conversation. Cultural consideration should be taken to avoid offending patients, since some HCPs reported that some Latina patients find anatomical diagrams to be offensive or embarrassing.

Our HCPs indicated that many adolescent Latinas with DM and their families were resistant to talking about RH-related topics. This is consistent with previous studies that indicate that RH-related communication is difficult due to some young Latinas’ discomfort about discussing sexuality with their mothers [28], their desire to please parents [29], and their reluctance to be forthright about sexual activity [29]. Prior research involving HCPs stated that there is often an intergenerational and inter-acculturation conflict regarding RH-related services because of clashes between traditional versus Americanized ideas on accessing RH services [29]. To overcome ambivalence in discussing sensitive topics such as birth control, PC and RH education, our HCPs suggested framing PC and RH information in terms of necessary knowledge for the future when the adolescent is married and ready to start her family, rather than suggesting that the teen may be currently sexually active. This may help parents to feel that their teens are not personally being targeted for current sexual activity. Prior studies indicate that the concept of virginity is especially important in Latino sexual values and beliefs, which may affect RH-related communication between the Latina adolescent and her partner(s) [30], as well as the Latina adolescent and her family [28,29,31]. Often, Latino adolescents believed that parents disapproved of their access to RH-related services because of the high value placed on virginity before marriage, the belief that RH-related services are for pregnant or promiscuous women only, and that RH-related conversations encourage sexual activity and promiscuity [29]. A qualitative study of young Latinas’ perceptions of parental approval for RH-related services found that most participants did not perceive parental support for RH-seeking behaviours [29].

Our study found that religion played a role in this population’s healthcare decision-making. Our HCPs suggested that providers consider assessing the family’s religious beliefs and, and if appropriate, take this into consideration when framing their message. Previous research indicates that a HCP’s selfdescription of being religious/spiritual, as well as the HCP’s comfort in asking about the patients’ religious beliefs, are associated with greater attention to spirituality and religion in clinical practice [32]. Furthermore, HCPs with more cross-cultural experiences report being better prepared to care for families whose religious beliefs impact clinical decision-making [18]. In our study, HCPS believed that including religion and faith when making healthcare decisions was well-received among Latino families. This perception was echoed in the Latina Teen Pregnancy Prevention Workgroup, which described religion as a cultural tension and a competing cultural demand [33]. Our HCP panel recommended approaching birth control in a careful and sensitive way because suggesting that adolescents take birth control could deeply offend adolescents and their parents [33]. High levels of religiosity, however, should not be assumed based on ethnic or cultural background.

The ADA recommends that all females of childbearing age receive education about the risks of maternal-child complications secondary to poor glucose control, as well as the importance of effective contraceptive use to prevent unplanned pregnancy, beginning at puberty [1]. Our HCPs agreed on the utmost importance of providing PC to their adolescent Latina patients with DM in order to prevent adverse outcomes and preserve future fertility; however, they emphasized that providers must first asses patient and family readiness and provide information in a culturally-sensitive manner. In order to ease discomfort and assess curiosity about RH-related topics, our HCPs suggested introducing PC and RH-related conversations to their adolescent Latina patients and their families by stating that they had new information to share with them. HCPs suggested reintroducing the topic at a subsequent visit if the adolescent or family members appear to be uncomfortable or not ready for the information. However, if the family is open to the discussion, the provider should give PC information that would promote healthy RH behaviors and aim to prevent DM-related RH complications.

To be culturally sensitive, our HCPs suggested couching PC and RH-related discussions in terms of the adolescent’s future sexual relationship and reproduction after marriage, and how effectively managing DM is crucial to facilitating future sexuality and family planning. This theme was also related to the importance of being ready/temporality/planning for the future, especially when managing DM. In addition, HCPs suggested using open-ended questions that were framed in a larger context (ex., “What are you thinking about in terms of marriage, or pregnancy, or preconception at this point?”) and giving anticipatory guidance about DM-specific PC as the conversation unfolded. Furthermore, patients with DM who seem to be overwhelmed with PC and RH information may benefit from having topics broken down of over the course of many visits.

Healthcare access and other barriers have also been cited by both our HCPs and the literature as playing a role in health care quality and patient outcomes in those with DM. Latinos have been shown to have poor access to insurance and express fears about deportation if they seek emergency care, which highlights systemic barriers to optimal DM management and contributes to their increased risk of DM-related complications [34–36]. Qualitative studies on Latinas with DM suggest that additional personal obstacles to receiving DM care include language barriers, poor DM awareness and control, long and/or inflexible work hours, different cultural norms, and transportation difficulties [34,36–39]. Economic barriers related to managing DM is also more pronounced in Latinos [37], with almost one in four reporting that they underuse their DM medications for financial reasons [40]. Some research suggests that healthcare access and low parental English proficiency are closely intertwined and severely impact many aspects of wellbeing for children with special healthcare needs, including the lack of regular provider or usual source of care [41,42].

Incorporating education about PC and DM-related RH health complications can be enhanced by incorporating a supportive network of people (both within and outside of the family network) to provide knowledge and guidance related to their DM and PC/RH. For example, *promotoras* (defined as a community health worker and a member of the community who work as bridges between HCPs and their ethnic, cultural, and geographical communities) can help to overcome individual and community barriers for adequate self-care and provide sensitive health care information [43]. Other studies have cited the importance of *promotoras* in increasing information about RH and decreasing barriers in Latinos [23].

To provide family-centered care, HCPs stated that stated that family-based sessions are useful in facilitating trust. These sessions may benefit from including extended family members (e.g., grandmothers) in the discussion, especially if these individuals will be making decisions about the adolescent’s care. However, matters of the daughter’s consent and privacy were not discussed in our panel.

Other studies suggest that young Latinas and HCPs perceive that family, particularly mothers, play an important role in young women’s access to RH-related services [29]. A study among 58 practitioners working with Latina adolescents in pregnancy prevention programs in California cited the cultural importance of strong family bonds and have recommended reinforcing this attribute in clinical practice [33]. Previous research indicates that older siblings have a high level of influence on younger siblings RH and sexual decision making in first and second-generation Latino families through modeling, close and supportive relationships, advice, social learning methods [44,45]. Although family-centered care appears to be valued by Latino youth, past research has indicated that there are lower odds of family-centered care for Latinos and for those who do not speak English as their primary language [46]. This disparity represents an important opportunity for improved cultural competence in the clinical setting.

Our HCPs also emphasized the importance of treating the teens as budding adults with their own autonomy. When HCPs were able to recognize and utilize the teens’ ability to make their choices as partners in health management, the teens gained respect for the providers and the care relationship was enhanced. By providing an open, ongoing dialogue regarding PC/RH information and incorporating previous themes of trust and autonomy, HCPs can empower adolescent Latinas to effectively manage their DM, be proactive in their RH-related decisions, and initiate discussions regarding RH and PC with their families, partners, and other providers. Prior qualitative studies indicate that adolescents’ feelings of personal autonomy is a major factor in their comfort and willingness to discuss RH with providers [47]. Encouraging a high level of autonomy and self-efficacy may have implications for future health.

## Limitations

There are several limitations to this qualitative study. First, many of these conversations focused on mother-daughter dyads in which the mother is a first-generation immigrant and the daughter is a second-generation immigrant. There was limited conversation on the dynamic in which both the mother and the daughter are first generation or second generation. Secondly, the HCPs were not always able to give an explanation of the cultural experiences that they perceived (ex., whether religious objections to PC-related conversations were due to a particular faith tradition, etc.). Thirdly, we acknowledge that Latinos represent a very diverse set of people, and that there are distinct differences among ethnic groups within this designation. Our HCPs did not make definitive distinctions between subgroups of Latinos, and our HCPs’ views are unlikely to accurately represent all cultures in the Latino diaspora. Lastly, these are perceptions of the HCPs about mother-daughter dyads and may not accurately reflect the experiences of the patient and her family.

## Conclusion

The above recommendations should be incorporated in tailoring educational and care delivery programs. HCPs should be culturally sensitive and consider tailoring their counseling when caring for this population, especially when discussing RH and PC. Future research efforts should be directed toward developing PC programs that are culturally sensitive and relevant for adolescent Latinas with DM.

## Figures and Tables

**Table 1.** Open-Ended Questions.

What were your experiences like in caring for Latina teens? What were your challenges and opportunities?
What was your experience in providing preconception counseling to Latina teens?
How involved are the mothers when preconception counseling is provided?
How do you feel about involving the mothers, as well as the fathers, in preconception counseling? What experience have you had in this?
What kinds of things can we modify for our preconception counseling intervention to make it more culturally sensitive?

**Table 2.** Cultural Knowledge and Experiences Themes Identified.

Themes	Quotes
**1) Issues of Identity**	• “Latino vonth want to blend in with their peers. The last thing that thev want to do is stand out.”
	• “It’s a challenge to provide care for any youth, but especially Latino youth (due to) problems with their identity.”
	• “We see Latino adolescents speaking English more than Spanish because they want to look like their peers.”
	• “They (the Latina adolescent patients) don’t want to be different.”

**2) Acculturation**	• “Acculturation is more than generational differences. The longer you have been in this country, the higher the chance of acculturation.”
	• “When teenagers come into the clinic with their parents, it’s not a custom for parents (for the HCP) to speak with them (the adolescents) alone. Latino teenagers in the general population and their families are used to this. Asking first-generation parents to leave the examination room creates tension in the relationship.”
	• “When parents stay in the room, your opportunities to even discuss (RH and PC) become muted in that it’s hard to expand on topics that you would like. It has to do with generation, but it also has to do with acculturation”
	• “With the parents, all social aspects of their lives and other problems come to the visit when you see them. It’s more noticeable in the parents than in the teenagers. The teenagers may not bring up all of those things (social aspects that impact care) as openly as the parents until they know you much better.”

**3) Stigma**	• “Teens face a lot of self-image challenges where they are comparing themselves to others. When you’re dealing with children who have obesity, they may often be bullied or may be bullying others. They may be looking at others and want to be thinner or they may be looking at others and want a different shape.”

**4) Ambivalence Toward Birth Control, RH Education and PC Knowledge**	• “I think one of the stark differences between Latino populations and other populations is the lack of wanting to use birth control. There are thoughts that are not non-accurate but that may not be as accurate with the current medical understanding of how contraception works.”
	• ’s a huge difference between what this generation in the US feels about planning and about when childbearing should start compared to the parents and grandparents. It becomes difficult to have these conversations because there are such stark differences between what the adolescents feel about contraception and preconception versus what the parents and grandparents, second and third generation feel about it.”

**5) Cultural Sensitivity vs. Best Practice**	• “Introducing contraception for the management of polycystic ovarian syndrome or a heavy period—aside from family planning—is often cut off, especially based on a religious perspective.”


**Table 3.** Barriers Identified.

Themes	Responses
**1) Language Barriers**	• “If they can relate to you, especially if you speak the same language, adherence is better.”
	• “They (the parents) cannot blend as easily as the teens within the general population because most of them don’t speak English.”

**2) Impact of Religion**	• “Religion plays a role. The parents and grandparents follow religion a little bit more. They’re a little bit more aware of what the church may say, while the younger generations are a little bit more open. I’ve had instances of conflict when you try to talk about sexual and reproductive health.”

**3) Access to Healthcare**	• “Many Latino youth experience barriers to healthcare access, insurance, etc.”
	• “They (adolescents) probably choose not to go specialty clinics because they want to blend in, except if they have other barriers to healthcare access such as lack of insurance or maybe cultural competence.”

**4) Discomfort**	• “If the kid unfolds into a giggly mess, you should probably just move on at that point because the child and family are uncomfortable. It is definitely driven by the family unit and by the child themselves because they are not going to listen to what we say about preconception counseling unless they are in that contemplation stage of listening to the material.”
	• “I think having materials make the conversation a little bit easier to start. You can say, “Oh, we have new information to share!” and sort of give the reason to bring it up when parents may not otherwise bring it up.”


**Table 4.** Facilitators Identified.

Themes	Responses
**1) The Importance of Support and Support Networks**	• “Using *promotoras* helps to promote trust. That is something that we continue to do and it seems like people responds to that.”
	• “After bringing in young Hispanic college students to interact with our teens, we had one of the highest retention rates (in our clinical trial).”
	• “The *promotora* model helped us to provide education and seemed to work very well.”

**2) Promoting Trust Between HCPs,** **Daughters, and Families**	• “I think that trust and listening to not just the diabetes, but everything that goes with their social issues (is important).”
	• “I feel trust is very important.”

**3) Assessing Emotional Development**	• “Every child is different. Sometimes, (medical) residents will come back from an assessment and say, ‘You know, I don’t know that I feel that comfortable talking about sex with this child because they seem so young,’. I think that, at times, there’s the aspect of ‘Okay, so they may seem young, but they’ll still have questions,’ and the added aspect of ‘Maybe they seem young and really are too young to absorb this information.’”
	• “As my patients have gotten much older, they always tease me because they tell me everything I did (discussing sex and RH) was—even though I thought it was early, it was late.”
	• “Recognizing teens’ autonomy and that they are trying to gain that independence and their role in addressing their own health and health behavior… was probably the greatest factor supporting their follow-up.”

**4) Empowerment**	• (On making decisions that support a healthy pregnancy) “I think it’s very important that they feel empowered and making them feel that they have the power to decide these things and learn more.”
	• “The concept of really, really empowering these girls, and you do a great job on what you have and when you say, ‘You can do it when the time is right. You can have a healthy pregnancy,’ and then somewhere else you say, ‘Choice, you have the power to choose.’”

**5) Safety**	• “Allow them to be safe with their diabetes and to think about the future so that it may motivate them.”
	• “When they get older—close to 17 or 18—I start talking about the importance of being safe during pregnancy, having the target range of blood sugars, and knowing where you are in your diabetes management.”

**6) Communicating in Patients’ Preferred Language**	• “Sometimes, splitting and speaking in English to the adolescent and Spanish to the parent has been really helpful in facilitating the interrelationship with us as a team with the parent and the child.”
	• “If you speak the same language, adherence is better.”
	• “Address the child in the language they want to speak.”

**7) Discussing RH-Related Topics and PC Using Cultural Sensitivity**	• “Mention marriage. Find some way to work the story around marriage and sex and repro-ducing as part of a package.”
	• “I wouldn’t presume to speak on what the role of religion or their faith position is, but ask them if it is important enough to mention.”
	• “It’s okay to talk about morals.”

**8) Importance Being Ready/Temporality/Planning for the Future**	• “I say that we are giving this to you in preparation for the future. It makes is easier for families to accept the information versus it being something like, ‘No, this doesn’t apply to her, because she’s not having sex.’”
	• (On framing RH-related conversations): “We frame the message as one thing that could be helpful, because we want to get to an individual as early as we can so that they can get information that can benefit them in the future; then, the parents look at this not as if you are labeling their children who have increased sexual activity but that you want to prevent challenges with pregnancy and childbirth as early as possible. This is designed to generate knowledge versus feeling like they are being targeted.”

**9)Importance of Family-Centered Care**	• “The more you involve the extended family, the better.”
	• “With some of our patients, the grandparents are actually the ones making all the decisions. The parents are there and involved in the discussion, but the final decision will come from the grandparents.”
	• “The girls come with mom and then they (the wife and daughter) have to talk to the dad before making decisions.”
	• “Dads want to know what is going on but always don’t want to know more. They can feel uncomfortable.”
	• “I have seen that often the mom has to go and talk to the dad. He’s like the main presence, even if he is not there (in the clinic). So, make them (the Latina adolescents) feel that they have the power to decide these things and learn more.”

**10) Variation in Educational Tailoring and Dissemination/ Care Delivery**	• “I would break down (RH information and PC) into different sections over a year, but those with different education may have a little trouble with it.”
	• “Some of my first-generation patients would say, ‘There is a picture of a uterus…gets it out of my face!’”
	• “In our endocrine clinic, we deal with puberty, breasts, gonads, penises, and we have pic-tures on our wall. Those pictures are now covered in dresses because part of our Hispanic population was very much offended by those pictures and complained to administration.”
	• “I would point out the difference between the first generation coming from Managua or Mexico City, and first generation coming from really rural areas.”

